# *Neisseria bacilliformis* is a periodontal pathogen exacerbating periodontitis by inducing nitric oxide production

**DOI:** 10.3389/fimmu.2025.1735500

**Published:** 2026-01-05

**Authors:** Bo-Min Kim, Yeonjin Lim, Somin Park, Jintaek Im, Cheol-Heui Yun, Kee-Yeon Kum, Ok-Jin Park, Seung Hyun Han

**Affiliations:** 1 Department of Oral Microbiology and Immunology, and DRI, School of Dentistry, Seoul National University, Seoul, Republic of Korea; 2 Department of Agricultural Biotechnology, and Research Institute of Agriculture and Life Sciences, Seoul National University, Seoul, Republic of Korea; 3 Department of Conservative Dentistry, Dental Research Institute, Seoul National University Dental Hospital, Seoul National University School of Dentistry, Seoul, Republic of Korea

**Keywords:** inflammatory response, lipooligosaccharide, *Neisseria bacilliformis*, nitric oxide, periodontitis, toll-like receptor 2, toll-like receptor 4

## Abstract

Periodontitis, a chronic inflammatory disease, often causes alveolar bone loss. *Neisseria bacilliformis* is a Gram-negative bacterium that has been identified in periodontal patients, but its role in periodontitis remains unclear. In the present study, we examined whether *N. bacilliformis* exacerbates periodontitis in a mouse ligature-induced periodontitis (LIP) model and investigated its underlying molecular mechanism. Topical treatment with *N. bacilliformis* on maxillary second molar exacerbated alveolar bone loss and worsened epithelial and periodontal ligament damage. Histological analyses showed that *N. bacilliformis* increases tartrate-resistant acid phosphatase (TRAP)-positive osteoclasts and inducible nitric oxide synthase (iNOS) levels in the gingival tissue. Treatment with *N. bacilliformis* induced nitric oxide (NO) production in RAW 264.7 cells, which was inhibited by polymyxin B, implying that *N. bacilliformis* lipooligosaccharide (LOS) is a major etiologic agent in the inflammatory response. Indeed, LOS purified from *N. bacilliformis* enhanced NO production and iNOS expression, primarily with activating Toll-like receptor (TLR) 4 and partially activating TLR2. LOS administration into the disto-palatal papilla near the molar further aggravated periodontitis in the LIP mouse model. These results suggest that *N. bacilliformis* is a periodontal pathogen that exacerbates inflammation and alveolar bone loss, with its LOS acting as an important virulence factor via TLR2/4 activation, leading to the production of the inflammatory mediator NO.

## 1 Introduction

Periodontitis is a chronic inflammatory oral disease caused by periodontopathic bacteria, leading to destruction of the periodontal ligament and alveolar bone loss (1). Socransky et al. analyzed subgingival plaque samples from patients with periodontitis and classified bacterial species into five major groups based on the severity of periodontitis (2). Among these, *Porphyromonas gingivalis*, *Tannerella forsythia*, and *Treponema denticola*—the most common bacteria found in individuals with severe periodontitis—are classified as red complex bacteria, with *P. gingivalis* recognized as the keystone pathogen. The overgrowth of pathogenic bacteria disrupts the balance of the microbiota in the subgingival pocket (3), leading to periodontal ligament destruction, periodontal pocket deepening, and alveolar bone loss (4). Recently, next-generation sequencing (NGS) technologies have continued to uncover novel disease-associated bacteria, including *Neisseria bacilliformis* (5). Understanding the pathogenesis of diseases caused by these newly identified periodontitis-associated bacteria is essential for diagnosis, prevention, and treatment of periodontal disease.

*N. bacilliformis* is an obligate aerobic, Gram-negative bacterium commonly found in the mucous membranes of mammals (6). Although typically a commensal bacterium, it has been reported as an opportunistic pathogen associated with oral and systemic diseases, including endocarditis (7). While present in smaller proportions compared to other bacteria, *N. bacilliformis* has been detected in the subgingival plaque of patients with chronic periodontitis (5). It has also been identified in the supragingival dental plaque of patients with severe early childhood caries, where its levels decreased following treatment (8). These findings suggest that *N. bacilliformis* is positively correlated with various oral diseases, including periodontitis and dental caries. However, while *N. bacilliformis* appears to be associated with oral diseases, its specific role in the disease development remains unclear.

Most Gram-negative bacteria possess lipopolysaccharide (LPS) with lipid A, a core oligosaccharide, and an O-antigen, a major virulence factor found in the outer membrane. The O-antigen is composed of repeating polysaccharide units containing up to eight glycosyl residues, which can repeat up to 50 times, resulting in diverse chain lengths (9). However, certain mucosal pathogens, such as *Neisseria* spp. and *Haemophilus influenzae*, have lipooligosaccharide (LOS) that lack the O-antigen (10). Both LPS and LOS activate Toll-like receptor 4 (TLR4), triggering the production of pro-inflammatory mediators such as tumor necrosis factor-α (TNF-α), interleukin-6 (IL-6), and nitiric oxide (NO) (11). NO is a reactive free radical molecule produced by NO synthase (NOS) through conversion of L-arginine to L-citrulline. NOS comprises three isoforms: endothelial NOS, neuronal NOS, and inducible NOS (iNOS). iNOS, primarily expressed in macrophages in response to LPS, LOS, and cytokine stimulation such as interferon-γ, plays a critical role in the inflammatory immune response (12). Elevated levels of iNOS in periodontal tissue result in increased NO production, which contributes to tissue destruction and bone resorption (13). Therefore, in this study, we investigated whether *N. bacilliformis* whole cell and its LOS directly contribute to the development of oral disease, specifically periodontitis, using a ligature-induced periodontitis (LIP) mouse model.

## 2 Materials and methods

### 2.1 Reagents and chemicals

Brain heart infusion (BHI) broth and bacto agar were obtained from BD Bioscience (San Diego, CA, USA). Chocolate agar was purchased from KisanBio (Seoul, Korea). Dulbecco’s modified eagle’s medium (DMEM) and Ham’s F-12 were purchased from Welgene (Daegu, Korea). Fetal bovine serum (FBS) was purchased from Gibco (Grand Island, NY, USA). Penicillin/streptomycin and trypsin-ethylenediaminetetraacetic acid (EDTA) were obtained from Hyclone (Logan, UT, USA). G418 and Hygromycin were purchased from Invitrogen (Thermo Fisher Scientific; Waltham, MA, USA) and Amresco (Solon, OH, USA), respectively. Recombinant murine macrophage colony-stimulating factor (M-CSF) and receptor activator of nuclear factor kappa-B ligand (RANKL) were purchased from R&D Systems (Minneapolis, MN, USA) and JW CreaGene (Seongnam, Korea), respectively. Polymyxin B sulfate salt (PMB), LPS from *Escherichia coli* O111:B4, and Nω-Nitro-L-arginine methyl ester hydrochloride (L-NAME) were purchased from Sigma-Aldrich (St. Louis, MO, USA). Tartrate-resistant acid phosphatase (TRAP) staining kit and mouse and rabbit specific horseradish peroxidase (HRP)/3,3’-Diaminobenzidine (DAB) detection immunohistochemistry (IHC) kit were purchased from CosmoBio (Tokyo, Japan) and Abcam (Cambridge, UK), respectively. Antibody specific to iNOS (#SC-7271) and rabbit polyclonal antibody against iNOS (#06-573) were purchased from Santa Cruz Biotechnology (Dallas, TX, USA) and Upstate Biotechnology (Lake Placid, NY, USA), respectively. Goat anti-rabbit IgG-HRP and anti-human CD25 IgG1-APC (#302610) were purchased from Southern Biotech (Birmingham, AL, USA) and Biolegend (San Diego, CA, USA), respectively. Coomassie Brilliant Blue R-250 staining and destaining solutions were obtained from Biosesang (Yongin, Korea).

### 2.2 Preparation of heat-killed *N. bacilliformis* and LOS

*N. bacilliformis* KCTC 23360 (Korean Collection for Type Culture; Daejeon, Korea) was cultivated in BHI at 37°C with shaking for 6 h. After harvesting, to prevent uncontrolled bacterial growth or spread in the macrophage cell culture systems, the bacterial pellets were washed with phosphate-buffered saline (PBS) and subsequently heat-inactivated at 70°C for 1 h. To confirm complete inactivation, the heat-inactivated *N. bacilliformis* was cultured on a BHI agar plate, and the absence of bacterial colony formation was verified (data not shown). *Neisseria meningitidis* serogroup B American Type Culture Collection (ATCC) 13090 was kindly provided by Professor Jeon-Soo Shin (Yonsei University, Seoul, Korea) and used to demonstrate that the LOS of *N. bacilliformis* shares the same structural forms. The strain was grown in chocolate agar at 37°C in a jar for 6 h. LOS from *N. bacilliformis* and *N. meningitidis* were extracted using the LPS extraction kit (iNtRON Biotechnology; Seongnam, Korea) according to the manufacturer’s instructions. The isolated LOS was quantified using the Pierce™ chromogenic endotoxin quant kit (Thermo Fisher Scientific; Waltham, MA, USA).

### 2.3 Animals

Animal experiments were conducted under approval of the Institutional Animal Care and Use Committee of Seoul National University (SNU-211018-5-3). C57BL/6 mice were obtained from Dooyeol Biotech (Seoul, Korea). C57BL/6 background TLR2- and TLR4-deficient mice were provided by Professor Shizuo Akira (Osaka University, Osaka, Japan). All mice were maintained under specific pathogen–free conditions.

### 2.4 LIP mouse model

LIP was induced in 9- to 10-week-old male C57BL/6 mice. All animals were randomly assigned to one of the following groups (*n* = 5/group): control, LIP, LIP + live *N. bacilliformis*, and LIP + *N. bacilliformis* LOS. A 5–0 soft silk ligature was tied around the maxillary second molar and remained in place throughout the experimental period in all mice. Except for the first day when the ligature was placed on maxillary second molar, isoflurane anesthesia was used for all procedures. For the *N. bacilliformis* treatment group, *N. bacilliformis* (1 × 10^9^ CFU) with 1.5% carboxymethyl cellulose (CMC) was applied to the silk ligature every other day for a total of three treatments. In a separate experiment, *N. bacilliformis* LOS (5 or 20 μg/4 μl) was injected into the disto-palatal papilla on both sides of the right second molar (2 μl per site) using an insulin syringe with a 30G needle (Seongshim Medical Co., Anyang, Korea). Mice were treated around the ligature placement on the second molar with *N. bacilliformis* (1 × 10^9^ CFU) or 1.5% CMC (n = 5). The sample size (*n* = 5) was determined based on previous ligature-induced periodontitis studies in mice, where n = 5 per group has been commonly used and shown to be sufficient to detect statistically significant differences in alveolar bone loss (14, 15).

### 2.5 Micro-computed tomography analysis

Seven days post-ligature placement, the mice were anesthetized and euthanized via cardiac perfusion with PBS. The molars were harvested and fixed in 4% paraformaldehyde overnight and then with PBS. Micro-CT scanning (Skyscan 1272; Bruker, Kontich, Belgium) was performed, and three-dimensional images were generated using CT-Volume software. Bone loss was assessed by measuring the distance from the cemento-enamel junction (CEJ) to the alveolar bone crest (ABC) using ImageJ software (National Institutes of Health, Bethesda, MD, USA). Alveolar bone loss was measured as the distance from the CEJ to the ABC. Measurements were taken at four sites per molar (third mesial root, distal and mesial roots of the second and first molars), and the mean CEJ–ABC distance was calculated for each group.

### 2.6 Histological analysis

The maxillae were decalcified in 10% EDTA (pH 7) at 4°C for two weeks. After decalcification, the teeth were embedded in paraffin, positioned with the buccal side facing downward, and sectioned at a thickness of 5 µm. The sections were deparaffinized, rehydrated, and stained with hematoxylin/eosin (H&E) and TRAP, as previously described (16). For H&E staining, inflammatory cell infiltration was semi−quantitatively scored (0-3) based on the extent and density of inflammatory cell accumulation within the subepithelial and gingival connective tissue (17). For TRAP staining, number of osteoclast (N.Oc) and bone surface (BS) were quantified as N.Oc/BS using Image J. For each sample, regions of interest were defined in the alveolar bone between the maxillary first and second molars (M1–M2) and between the second and third molars (M2–M3). Bone surface (BS) and osteoclast numbers were measured in these regions, and osteoclast counts were normalized to BS (18). iNOS expression in gingival tissue was detected by immunohistochemistry (IHC) using an anti-iNOS antibody and an HRP/DAB detection kit according to the manufacturer’s protocol. iNOS immunostaining was quantified using the IHC Profiler plugin in ImageJ. H−scores were calculated from IHC−Profiler output as H−score = 3 × (% high positive) + 2 × (% positive) + 1 × (% low positive) for each region of interest (19). Images were captured using a digital upright fluorescence microscope (Olympus Corporation, Tokyo, Japan).

### 2.7 Cell culture

RAW 264.7 cells were obtained from the ATCC (Manassas, VA, USA) and cultured in DMEM containing 10% heat-inactivated FBS, 100 U/ml penicillin, and 100 μg/ml streptomycin at 37°C in a humidified incubator with 5% CO_2_. The cells were seeded into 96-well plates at 200 μl of 3 × 10^5^ cells/ml/well and stimulated with the indicated stimuli for 24 h. In a separate experiment, bone marrow-derived macrophages (BMMs) were prepared as described previously (20). These cells were seeded into 96-well plates at 200 μl of 3 × 10^5^ cells/ml/well and stimulated with the indicated stimuli for 24 h. To examine osteoclast differentiation, BMMs were stimulated with 30 ng/ml RANKL and 30 ng/ml M-CSF for 2 days to generate committed osteoclasts. The committed osteoclasts were subsequently treated with *N. bacilliformis* LOS (3 or 30 ng/ml) for 22 h, and osteoclast activity was evaluated by TRAP staining.

### 2.8 NO assay

NO production was analyzed by measuring nitrate levels in the supernatant. Equal volumes of Griess reagent (1% sulfanilamide, 0.1% naphthylethylenediamine dihydrochloride, and 2% phosphoric acid) and the supernatant were mixed and incubated at room temperature for 5 minutes. Nitrate levels were measured at 540 nm using a microplate reader (Tecan Group Ltd., Männedorf, Switzerland), with NaNO_2_ used as a standard.

### 2.9 Coomassie blue staining and silver staining

*N. bacilliformis* LOS, *N. meningitidis* LOS, and *E. coli* LPS were heated at 100°C for 5 min, loaded into each well, and separated using 15% sodium dodecyl sulfate-polyacrylamide electrophoresis (SDS-PAGE) gel. The gel was stained with Coomassie Brilliant Blue R-250 staining solution for 1 h, followed by overnight de-staining (45% methanol/10% glacial acetic acid). Silver staining was then performed as described previously (21).

### 2.10 Reverse transcription-polymerase chain reaction

RAW 264.7 cells were seeded into 6-well plates at 2 ml of 3 × 10^5^ cells/ml/well and stimulated with the indicated stimuli for 6 h. The detection of iNOS mRNA was performed as described previously (22) with the primers specific to murine iNOS (forward primer: 5′-CGCTTGGGTCTTGTTCACTC-3′, reverse primer: 5′-GGTCATCTTGTATTGTTGGG-3′) or GAPDH (forward primer: 5′-ACCCAGAAGACTGTGGATGG-3′, reverse primer 5′-CACATTGGGGGTAGGAACAC-3′). Melt curve and amplicon plot analyses demonstrated a single sharp peak for each primer pair, with no evidence of nonspecific amplification, thereby confirming amplification of a single product of the expected size.

### 2.11 Western blot assay

RAW 264.7 cells were seeded into 6-well plates at 2 ml of 3 × 10^5^ cells/ml/well and treated with the indicated stimuli for 24 h. The cells were washed twice with PBS and lysed using RIPA buffer (LPS Solution, Daejeon, Korea). Western blotting was performed as described previously (23). Immunoreactive bands were detected using SUPEX ECL reagents (Neuronex, Daegu, Korea) and visualized with the Fusion FX6.0 imaging system (Vilber Lourmat, Marne-la-Vallée, France).

### 2.12 Measurement of TLR2 and TLR4 activation

TLR2 and TLR4 activation was assessed by detecting CD25 expression, as described previously (24). Briefly, CHO/CD14/TLR2 and CHO/CD14/TLR4 cells were seeded into 24-well plates at 500 μl of 8 × 10^5^ cells/ml/well and incubated for 9 h. The cells were then stimulated with the indicated stimuli overnight. CD25 expression was analyzed using flow cytometry (FACSVerse, Becton Dickinson, Franklin Lakes, NJ, USA).

### 2.13 Enzyme-linked immunosorbent assay

TNF-α and IL-6 were measured using ELISA kits, according to the manufacturer’s instructions (BioLegend).

### 2.14 Statistical analysis

The results are presented as mean ± standard deviation (S.D.) from at least three independent experiments unless stated otherwise. Statistical significance was examined by one-way ANOVA, followed by Tukey’s *post-hoc* test or Student’s t test using GraphPad Prism 6 software (GraphPad Software Inc., La Jolla, CA, USA) with significance (*) defined as *P* < 0.05.

## 3 Results

### 3.1 *N. bacilliformis* exacerbates periodontitis in the LIP mouse model

**Figure 1:**
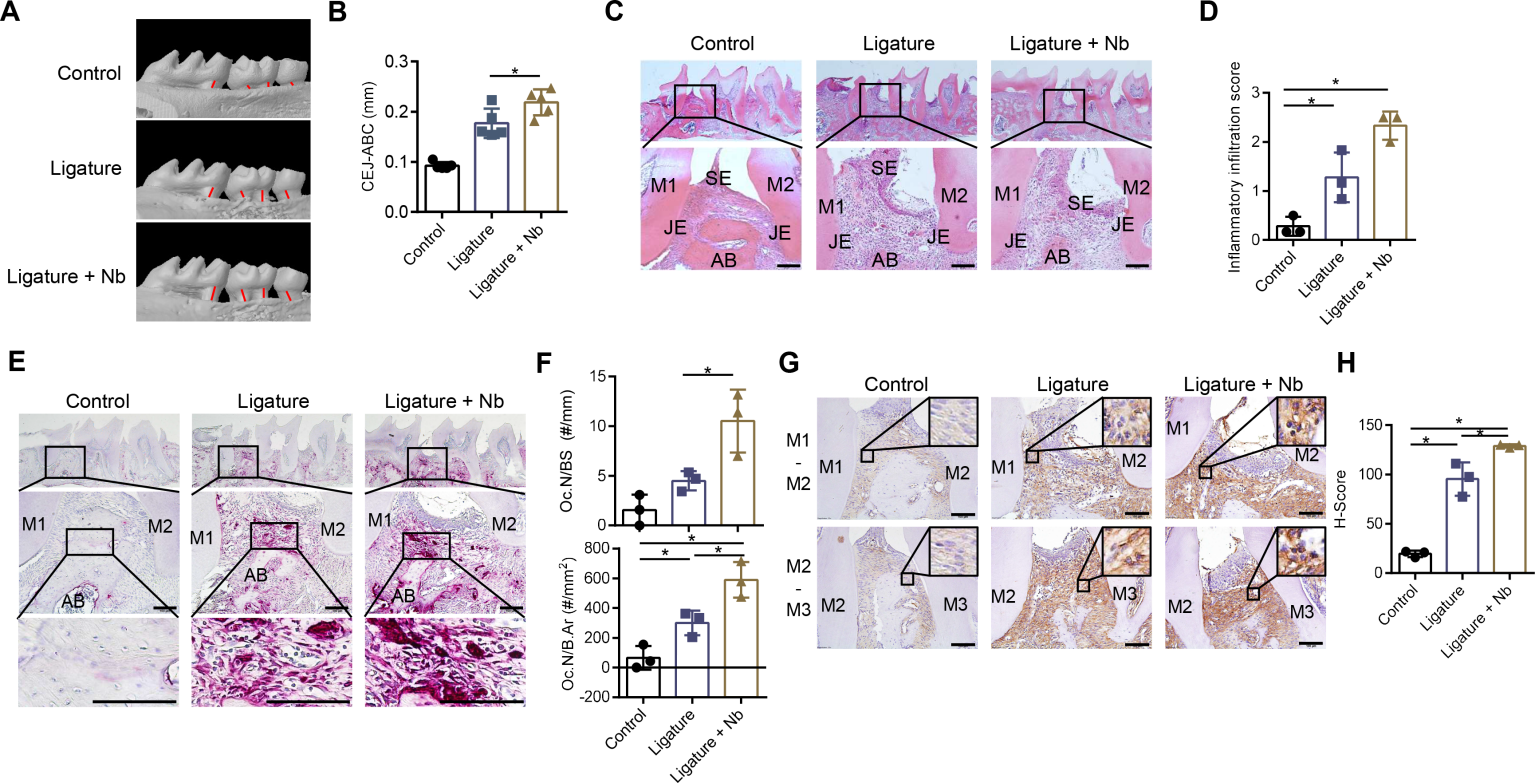
*N. bacilliformis* exacerbates periodontitis and upregulates iNOS expression. Mice were treated around the ligature placement on the second molar with live *N. bacilliformis* (10^9^ CFU) or 1.5% CMC (*n =* 5). After 7 days, micro-CT scans were performed on right maxillary molars. **(A)** Representative three-dimensional micro-CT images of maxillary molars from the control (non-ligatured, *n =* 5), ligature-only (treated with 1.5% CMC, *n =* 5), and ligatured with *N. bacilliformis* (10^9^ CFU, *n =* 5) mice. **(B)** Alveolar bone loss was quantified by measuring the distance (mm) from the cementoenamel junction (CEJ) to the alveolar bone crest (ABC) of the second molar. The red bar (panel A) indicates the CEJ-ABC distance. **(C)** H&E staining of the second molar. Scale bar = 100 µm. **(D)** Inflammatory cell infiltration score within the subepithelial and gingival connective tissue using ImageJ. **(E)** TRAP staining of gingival tissue near the second molar. Scale bar = 100 µm. **(F)** Osteoclast numbers and bone surface (BS) were quantified as N.Oc/BS using Image J. **(G)** IHC staining of secondary molar gingival tissue for iNOS. **(H)** iNOS immunostaining was quantified using the IHC Profiler plugin in ImageJ. Scale bar = 100 µm. H&E, Hematoxylin and eosin; TRAP, tartrate-resistant acid phosphatase; M1, molar 1; M2, molar 2; M3, molar 3; SE, surface epithelium; JE, junctional epithelium; AB, alveolar bone crest. *P< 0.05.

We investigated whether *N. bacilliformis* exacerbates periodontitis using a mouse LIP model. Micro-CT analysis showed that the average CEJ-ABC distance was increased in the live *N. bacilliformis*-treated ligature group compared to the non-treated ligature group (mean values of 0.218 and 0.176 mm, respectively) (**Figures 1A, B**). In tissue sections with H&E staining, the control group exhibited a triangular-shaped gingiva and thick alveolar bone. In contrast, seven days post-ligature placement, mice showed destruction of the junctional epithelium and periodontal ligament, along with alveolar bone resorption compared to the control group (**Figure 1C**). *N. bacilliformis*-treated ligature mice showed more severe gingival tissue destruction and deeper subgingival pockets (**Figure 1C**) along with an increased accumulation of inflammatory cells within the subepithelial and gingival connective tissue (**Figure 1D**). Furthermore, TRAP-positive cells around the alveolar bone were increased in the *N. bacilliformis*-treated ligature mice compared to the non-treated ligature group, indicating increased osteoclast activity (**Figures 1E, F**). Since elevated NO levels act as a positive regulator of tissue destruction and bone resorption (13), we examined the expression of iNOS in gingival tissue using immunohistochemistry. As shown in **Figures 1G, H**, iNOS expression was markedly increased in *N. bacilliformis*-treated ligature mice compared to both control and non-treated ligature groups. These findings indicate that *N. bacilliformis* exacerbates periodontitis through the upregulation of NO production.

### 3.2 Heat-killed *N. bacilliformis* induces NO production in macrophages

**Figure 2:**
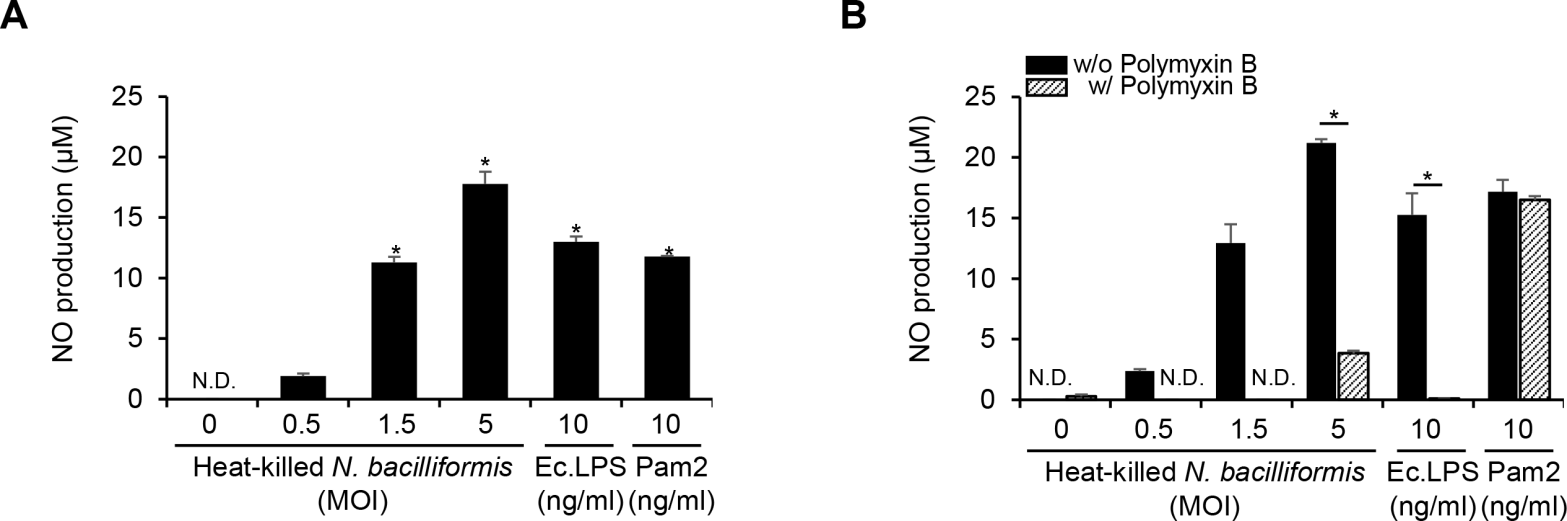
Heat-killed *N. bacilliformis* induces NO production in macrophages. **(A)** RAW 264.7 cells (3 × 10^5^ cells/ml) were stimulated with heat-killed *N. bacilliformis* at 0, 0.5, 1.5, or 5 MOI for 24 h. **(B)** RAW 264.7 cells (3 × 10^5^ cells/ml) were pre-treated with or without 25 µg/ml of polymyxin B sulfate for 30 min, followed by stimulation with heat-killed *N. bacilliformis*, *E*. *coli* LPS (Ec.LPS,10 ng/ml), or Pam2CSK4 (Pam2, 10 ng/ml) at the indicated concentrations for an additional 24 h. NO production was measured using the Griess reagent assay. *E*. *coli* LPS and Pam2CSK4 were used as positive controls for TLR4 and TLR2 activation, respectively. All results are expressed as the mean ± S.D. of triplicate samples. *P < 0.05., N.D., not detected.

Since NO is primarily produced by macrophages (25), we treated the murine macrophage cell line RAW 264.7 with *N. bacilliformis* to determine whether it induces NO production. As shown in **Figure 2A**, similar to *E. coli* LPS and Pam2CSK4 (mimicking bacterial lipoproteins), heat-killed *N. bacilliformis* increased NO production in a dose-dependent manner. Next, since LPS is a well-known potent virulence factor of Gram-negative bacteria, we examined whether the NO induction by *N. bacilliformis* was due to its LOS using a lipid A-targeting LPS blocker, PMB. Pre-treatment with PMB substantially reduced NO production induced by both *N. bacilliformis* and *E. coli* LPS but not that by Pam2CSK4 (**Figure 2B**). These results suggest that *N. bacilliformis* LOS might play a critical role in NO production in macrophages.

### 3.3 *N. bacilliformis* LOS induces NO production in macrophages

**Figure 3:**
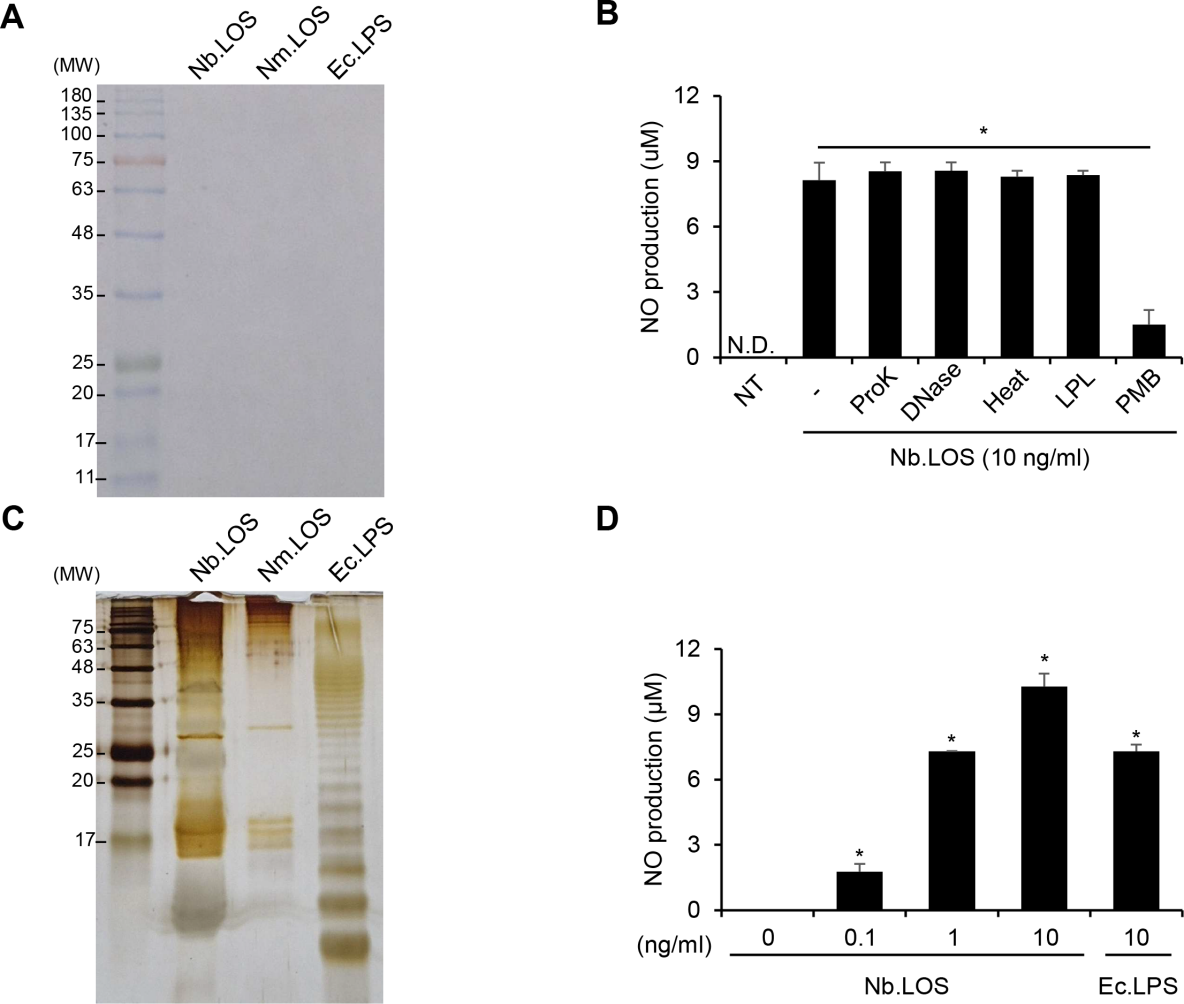
*Neisseria bacilliformis* LOS induces NO production in macrophages. **(A, B)***N. bacilliformis* LOS (Nb.LOS, 10 μg), *N. meningitidis* LOS (Nm.LOS, 10 μg), and *E*. *coli* LPS (Ec.LPS, 10 μg) were loaded onto a 15% SDS-PAGE gel and separated by electrophoresis. Molecular weight standards (M), Nb.LOS, Nm.LOS, and Ec.LPS were visualized by Coomassie blue staining **(A)** and silver staining **(C)**. **(B)** Nb.LOS was treated with proteinase K (ProK, 50 μg/ml), DNase I (DNase, 50 μg/ml), heat (100°C, 10 min), and lipoprotein lipase (LPL, 50 μg/ml), or polymyxin B sulfate (PMB, 25 μg/ml). RAW 264.7 cells (3 × 10^5^ cells/ml) were stimulated with 10 ng/ml of each treated Nb.LOS. **(D)** RAW 264.7 cells (3 × 10^5^ cells/ml) were stimulated with Nb.LOS at 0.1, 1, or 10 ng/ml or Ec.LPS at 10 ng/ml for 24 h. NO production was quantified using the Griess reagent assay. *P < 0.05., N.D., not detected.

To determine whether *N. bacilliformis* LOS induces NO production, we purified LOS from *N. bacilliformis*. As shown in **Figure 3A**, impurities in the LOS preparation was hardly detected in the Coomassie blue staining. When *N. bacilliformis* LOS was treated with Proteinase K, DNase I, heat, or lipoprotein lipase, NO induction remained unchanged compared to that of untreated *N. bacilliformis* LOS. However, PMB-treated *N. bacilliformis* LOS showed reduced NO production (**Figure 3B**), indicating NO induction by *N. bacilliformis* LOS was not due to impurities in the LOS preparation, but rather was caused by the LOS itself. Since *Neisseria* species are known to have LOS that lack the O-antigen (10), we determined whether *N. bacilliformis* LOS also lacks the O-antigen. In silver staining, the band pattern of *N. bacilliformis* LOS was similar to that of *N. meningitidis* LOS but distinct from that of *E. coli* LPS, which contains O-antigen ladders. Most of the bands were located in the lower region (**Figure 3C**). These results suggest that the endotoxin of *N. bacilliformis* is LOS, an O-antigen-deficient form of LPS. *N. bacilliformis* LOS increased NO production in a dose-dependent manner in RAW 264.7 cells (**Figure 3D**). Collectively, these findings indicate that LOS is the major factor responsible for NO induction by *N. bacilliformis* in macrophages.

### 3.4 *N. bacilliformis* LOS augments iNOS expression in macrophages

**Figure 4:**
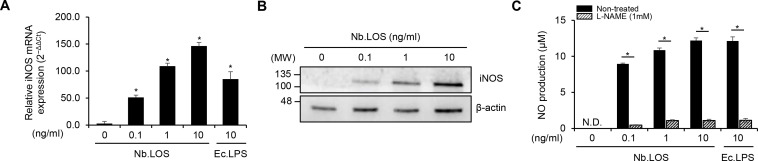
*N. bacilliformis* LOS augments iNOS-derived NO in macrophages. RAW 264.7 cells (3 × 10^5^ cells/ml) were stimulated with Nb.LOS at 0.1, 1, or 10 ng/ml or Ec.LPS at 10 ng/ml for 6 h **(A)** and 24 h **(B)**. **(A)** The mRNA expression level of iNOS was quantified using real-time RT-PCR. Relative expression level of each gene was normalized to the level of GAPDH. **(B)** The protein expression level of iNOS was determined by Western blot analysis. **(C)** RAW 264.7 cells (3 × 10^5^ cells/ml) were pre-treated with or without 1 mM of L-NAME for 1 h, followed by stimulation with Nb.LOS at 0.1, 1, or 10 ng/ml or Ec.LPS at 10 ng/ml for an additional 24 h. NO production was measured using the Griess reagent assay. *P< 0.05.

iNOS is a critical enzyme responsible for the production of large amounts of NO (25). Thus, we examined the expression of iNOS in macrophages treated with *N. bacilliformis* LOS. *N. bacilliformis* LOS increased iNOS mRNA expression in a dose-dependent manner in RAW 264.7 cells (**Figure 4A**, **Supplementary Table 1**). Consistent with this result, an increase in iNOS protein level was also observed (**Figure 4B**, **Supplementary Table 1**). In addition, pretreatment with L-NAME, an iNOS inhibitor, abolished *N. bacilliformis* LOS–induced NO production, indicating that NO induction by LOS is mediated through iNOS upregulation (**Figure 4C**). These results indicate that *N. bacilliformis* LOS enhances iNOS expression in macrophages, ultimately leading to elevated NO production.

### 3.5 *N. bacilliformis* LOS induces NO through TLR4 and partially through TLR2 in macrophages

**Figure 5:**
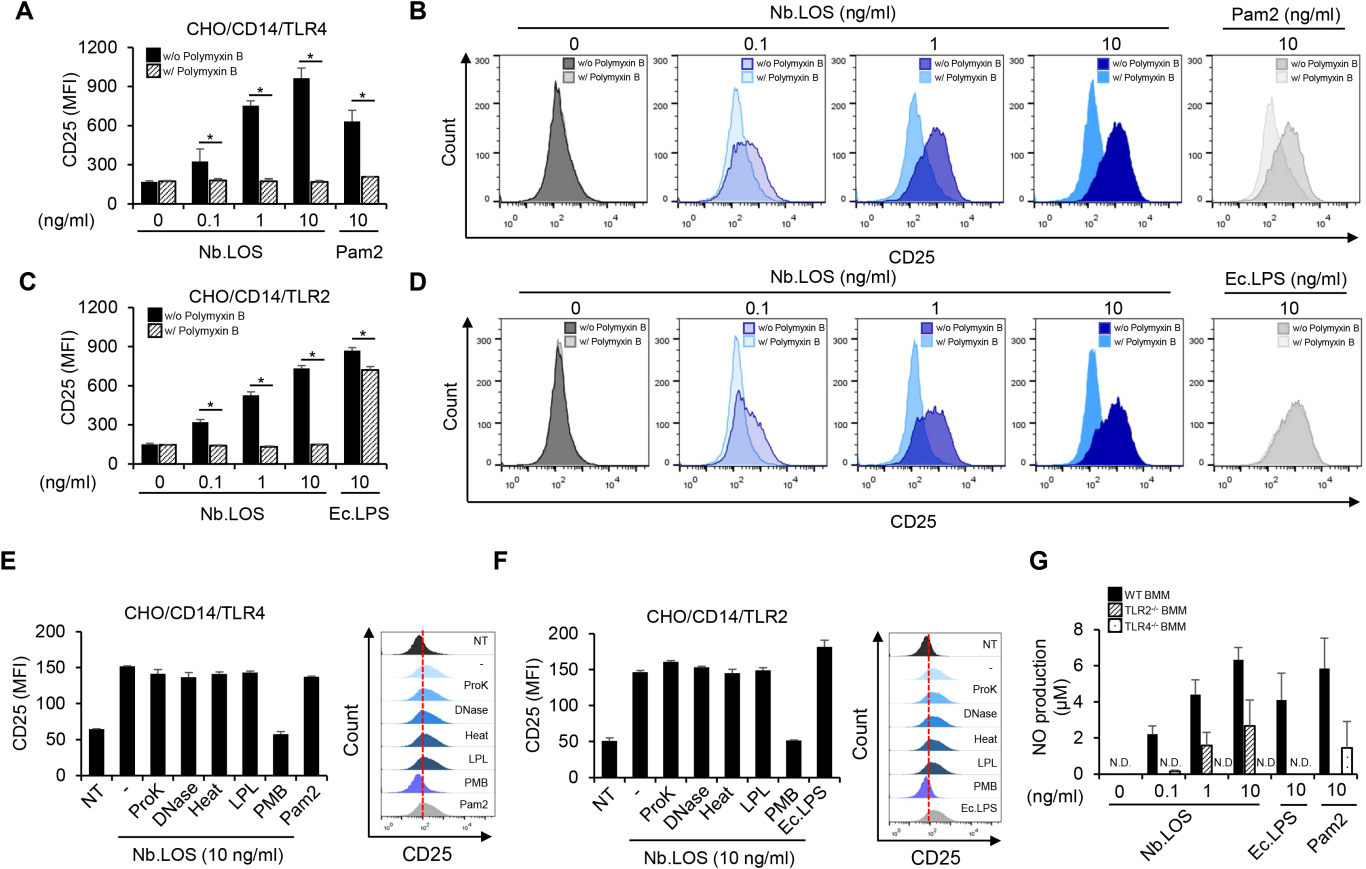
TLR2 and TLR4 are essential for induction of NO production in macrophages by *N. bacilliformis* LOS. CHO/CD14/TLR4 **(A, B, E)** and CHO/CD14/TLR2 **(C, D, F)** cells (6 × 10^5^ cells/ml) were pre-treated with or without 25 µg/ml of PMB for 30 min. The cells were then stimulated with the indicated materials for an additional 16 h. The expression of CD25, measured as mean fluorescence intensity (MFI), was analyzed by flow cytometry as a marker of TLR2- or TLR4-dependent NF-κB activation. **(G)** Wild-type, TLR2, or TLR4-deficient BMMs (3 × 10^5^ cells/ml) were stimulated with Nb.LOS at 0, 0.1, 1, or 10 ng/ml for 24 h. NO production was quantified using the Griess reagent assay. Pam2CSK4 (Pam2, 10 ng/ml) and *E. coli* LPS (Ec.LPS, 10 ng/ml) were used as controls for TLR2 and TLR4 activation, respectively. All results were expressed as the mean ± S.D. of triplicate samples. *P< 0.05.

Both LPS and LOS are known to be recognized by TLR4 (26, 27). We further examined whether *N. bacilliformis* LOS induces an inflammatory response through TLR4. As shown in **Figures 5A, B**, *N. bacilliformis* LOS induces CD25 expression in CHO/CD14/TLR4 cells in a dose-dependent manner. However, unexpectedly, CD25 expression was also observed in CHO/CD14/TLR2 cells (**Figures 5C, D**). In addition, when *N. bacilliformis* LOS was treated with Proteinase K, DNase I, heat, or lipoprotein lipase, *N. bacilliformis* LOS−induced CD25 expression was not significantly altered by these treatments, implying that the observed TLR2 activation is unlikely to be due to residual lipoprotein contamination. To clearly determine whether *N. bacilliformis* LOS activates both TLR2 and 4 signaling pathways, primary BMMs derived from wild-type, TLR2-deficient, or TLR4-deficient mice were treated with *N. bacilliformis* LOS. In TLR4-deficient BMMs, NO production was completely abolished, whereas in TLR2-deficient BMMs, it was reduced by approximately 50% (**Figure 5G**). These results suggest that *N. bacilliformis* LOS primarily induces an inflammatory response via TLR4 and partially through TLR2.

### 3.6 *N. bacilliformis* LOS aggravates periodontitis *in vivo*

**Figure 6:**
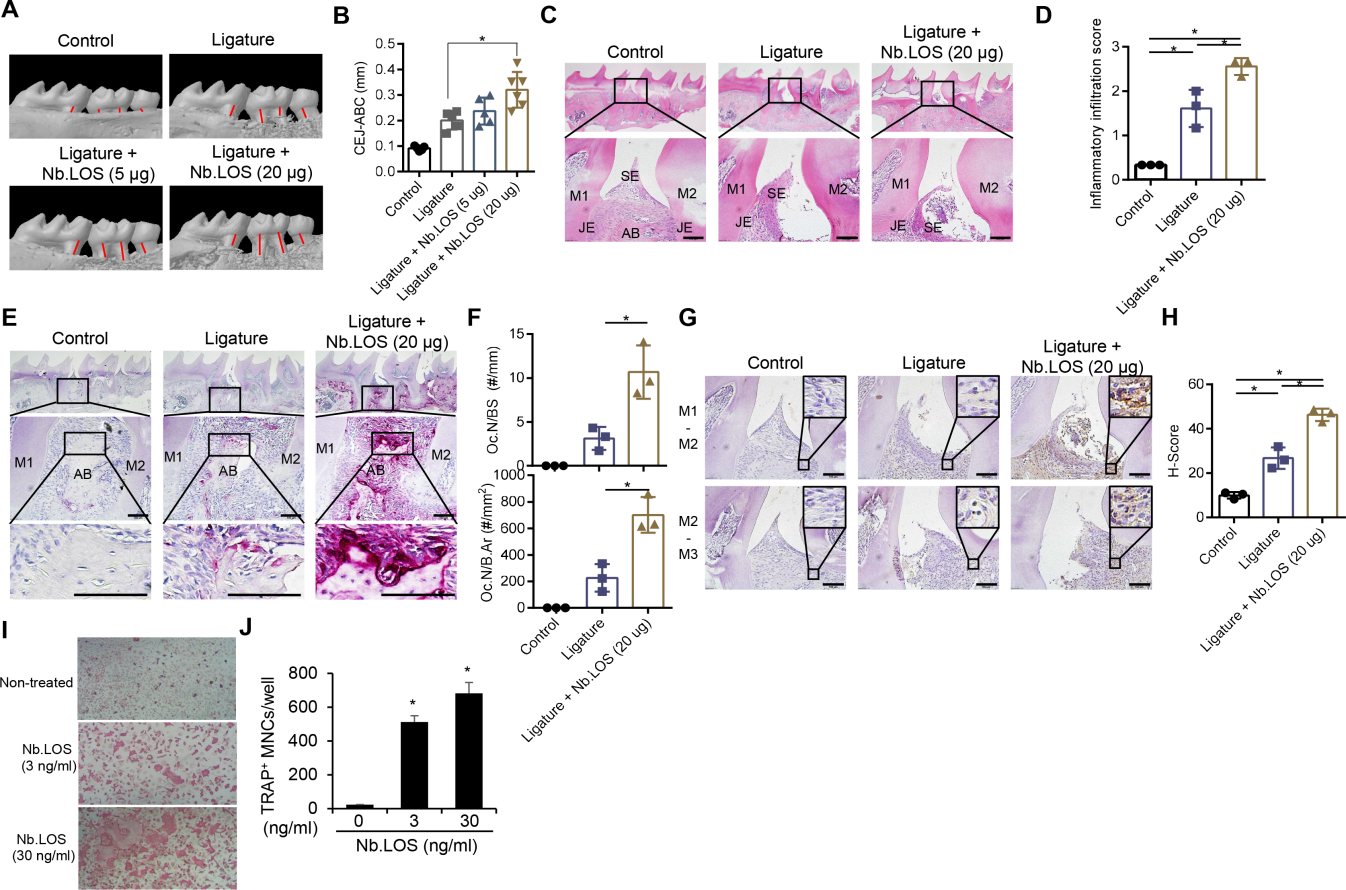
Nb.LOS aggravates periodontitis by upregulation of NO production and osteoclasts. Mice were injected with 5 μg of *N. bacilliformis* LOS (*n* = 5), 20 μg of Nb.LOS (*n* = 6), or endotoxin-free water (*n* = 5) into the disto-palatal papilla of both sites of the right second molar. After 7 days, micro-CT scans were performed on the right maxillary molars. **(A)** Representative three-dimensional micro-CT images of maxillary molars from control mice, mice ligatured with EFW (ligature), mice ligatured with 5 μg of Nb.LOS, and mice ligatured with 20 μg of Nb.LOS. The red bar indicates the cementoenamel junction (CEJ)-alveolar bone crest (ABC) distance. **(B)** Alveolar bone loss: distance (mm) from the CEJ to the ABC of the second molar was measured. **(C)** H&E staining of the second molar gingival tissue. Scale bar = 100 µm. **(D)** Inflammatory cell infiltration score within the subepithelial and gingival connective tissue using ImageJ. **(E)** TRAP staining of the second molar gingival tissue. Scale bar = 100 µm. **(F)** Osteoclast numbers and bone surface (BS) were quantified as N.Oc/BS using Image J. **(G)** IHC staining of secondary molar gingival tissue for iNOS. **(H)** iNOS immunostaining was quantified using the IHC Profiler plugin in ImageJ. Scale bar = 100 µm. M1, molar 1; M2, molar 2; M3, molar 3; SE, surface epithelium; JE, junctional epithelium; AB, alveolar bone crest. **(I, J)** Committed osteoclasts were stimulated with Nb.LOS (3 or 30 ng/ml) for 22 h, and osteoclast activity was evaluated by TRAP staining. TRAP-positive multinucleated cells with three or more nuclei were enumerated through microscopic analysis. *P < 0.05.

Since *N. bacilliformis* LOS efficiently induces NO, we investigated whether it enhances periodontitis development *in vivo*. As shown in **Figures 6A, B**, *N. bacilliformis* LOS increased the CEJ-ABC distance compared to the non-treated ligature group, indicating that *N. bacilliformis* LOS accelerates periodontitis progression. Furthermore, *N. bacilliformis* LOS exacerbated ligature-induced destruction of the junctional epithelium and inflammatory cell infiltration within the subepithelial and gingival connective tissue (**Figures 6C, D**). TRAP-positive osteoclast cells and iNOS expression were increased in the *N. bacilliformis* LOS-treated ligature group compared to the non-treated ligature group (**Figures 6E-H**). In addition, *N. bacilliformis* LOS induced TRAP-positive multinucleated osteoclasts in committed osteoclasts, indicating that *N. bacilliformis* LOS promotes osteoclast differentiation (**Figures 6I, J**). These results indicate that *N. bacilliformis* LOS plays a critical role in *N. bacilliformis*-accelerated periodontitis.

## 4 Discussion

The development of NGS technology enables the identification of novel disease-associated bacterial species, including unculturable or low-abundance bacterial species. *N. bacilliformis* has been identified in gingival plaque and reported to be associated with periodontitis by NGS analysis (5). However, its direct role in the pathogenesis of periodontitis has not been fully investigated. In this study, we demonstrated that *N. bacilliformis* exacerbates periodontitis by enhancing NO production, with its LOS acting as a key virulence factor in an LIP mouse model. These results suggest *N. bacilliformis* as a newly identified periodontopathic bacterium that contributes to the progression of periodontitis by promoting NO production in gingival tissue.

Our results demonstrated that *N. bacilliformis* aggravates periodontitis in a mouse LIP model. *N. bacilliformis* substantially increased the expression of TRAP around the alveolar bone and NO levels in gingival tissue compared to the non-treated ligature group. Similarly, periodontal pathogens such as *P. gingivalis* and *Fusobacterium nucleatum* have been shown to exacerbate ligature-induced periodontal bone resorption in the LIP mouse model (28, 29). Additionally, direct infection of *Aggregatibacter actinomycetemcomitans* into the palatal gingival tissue of the second molar results in alveolar bone loss (30). In contrast, multiple TM7 isolates, putative pathogens commonly found in periodontal plaque, have been reported to reduce inflammatory bone loss in the LIP mouse model (31). This finding suggests that since bacteria found in the plaque of periodontal disease patients may exhibit either pathogenic or beneficial properties, it is essential to clarify the pathogenic potential of the bacterial species. Of note, LIP mouse model is well-suited for studying tissue destruction and alveolar bone resorption, which are hallmark features of periodontitis (32). Periodontopathic bacteria, such as *P. gingivalis*, cause inflammation and alveolar bone loss, and *N. bacilliformis* demonstrated similar pathogenic effects in the current study. Therefore, *N. bacilliformis* might be a novel periodontopathic bacterium that contributes to the severity of periodontitis by promoting tissue destruction and bone resorption.

In our study, *N. bacilliformis* and its LOS efficiently induced NO production both *in vitro* and *in vivo*. Elevated levels of iNOS and NO play an important role in periodontal disease (33). For instance, iNOS expression is increased in the gingival tissue of periodontitis patients compared to healthy individuals (34). Also, NO synthase inhibitors exhibited reduction of alveolar bone loss in a rat LIP model (35). When *P. gingivalis* was inoculated into oral cavity of mice, wild-type mice exhibited alveolar bone loss, whereas iNOS-deficient mice were resistant to this effect (36). Although NO plays a role in host defense by protecting against pathogens (37), high concentrations of NO induced by an immunological signal such as LPS and inflammatory cytokines can trigger cytotoxic effects. These effects include inhibition of mitochondrial respiration, DNA damage, and protein modification, primarily mediated by highly toxic peroxynitrite anion (ONOO⁻), which forms from the reaction between NO and superoxide (O_2_⁻). Together, these mechanisms amplify the inflammatory response and ultimately lead to host tissue breakdown (37). Given that iNOS-derived NO is known to promote osteoclast activity and alveolar bone loss, our findings suggest that LOS contributes to periodontal bone resorption at least in part via iNOS-mediated NO induction. However, NO should be considered as one of several inflammatory mediators involved in this process, and further *in vivo* studies using iNOS inhibition or iNOS-deficient mice will be necessary to definitively establish the role of iNOS-derived NO in alveolar bone loss.

We showed that *N. bacilliformis* LOS, which differs from the typical LPS structure, plays a major role in NO induction. PMB, which neutralizes LPS/LOS, inhibited *N. bacilliformis*-induced NO production in macrophages. *N. bacilliformis* LOS accelerated periodontitis *in vivo*. In general, LPS/LOS, a potent virulence factor of Gram-negative bacteria, is generally recognized by TLR4 on host cells, such as macrophages. Activation of the MyD88-dependent pathway leads to activation of NF-κB, a transcription factor that induces inflammatory cytokines and mediators including NO (38). LPS derived from red complex bacteria, such as *P. gingivalis*, *T. denticola*, and *T. forsythia*, has been shown to induce the secretion of high levels of inflammatory cytokines in human whole blood from periodontitis patients (39). Furthermore, LPS has been reported to correlate with the development of diseases such as sepsis (40). Although our findings highlight NO induction by *N. bacilliformis* LOS, NO should be considered as one important contributor among several mechanisms leading to alveolar bone loss. Pro-inflammatory cytokines, reactive oxygen species (ROS), and osteoclastogenic signaling pathways are also likely to participate in this process (41–43). Thus, NO production represents part of a complex inflammatory cascade that collectively promotes osteoclast activity and bone resorption, thereby contributing to the development of periodontal disease.

TLR2 and TLR4 appear to play critical roles in *N. bacilliformis* LOS-induced NO production. In addition to NO induction, *N. bacilliformis* LOS also triggered secretion of pro-inflammatory cytokines such as TNF-α and IL-6 (**Supplementary Figure 2**), indicating that NO production occurs within a broader inflammatory network downstream of TLR2/TLR4 activation. Numerous studies have reported the importance of TLR2 and 4 signaling in the innate immune response to pathogens *via* NF-κB activation. In general, LPS is recognized by TLR4 (44). However, certain atypical LPS, such as those from *P. gingivalis* and *Ochrobactrum*, have been shown to activate immune responses through both TLR2- and TLR4-dependent pathways (45, 46). The acylation and phosphorylation pattern of lipid A could influence on the specificity of their recognition receptors. For instance, *P. gingivalis* LPS is highly heterogeneous, containing multiple lipid A forms, including tetra- and penta-acylated structures (45). These lipid A structures are found at high and low hemin concentrations, respectively (47). Due to this heterogeneity, tetra-acylated *P. gingivalis* LPS activates TLR2, whereas the penta-acylated form can activate both TLR2 and TLR4. Some researchers have suggested the possibility of activation triggered by impurities such as lipoprotein in LOS preparation (48). However, in the present study, *N. bacilliformis* LOS pre−treated with lipoprotein lipase, heat, and proteinase K still induced NO production and TLR2 activation, suggesting that lipoproteins are unlikely to account for the observed effects. Nevertheless, further studies will be required to elucidate the precise structural interactions of LOS with TLR2 and TLR4.

In conclusion, we identified *N. bacilliformis* as a periodontopathic bacterium that exacerbates the severity of periodontitis. Its LOS was a key molecule promoting NO production by stimulating primarily TLR4 and partially TLR2. Targeting NO induction or its related signaling pathways may offer an effective strategy for treating or preventing periodontal diseases caused by pathogens such as *N. bacilliformis*.

## Data Availability

The raw data supporting the conclusions of this article will be made available by the authors, without undue reservation.
