# Supplementary material for: Neisseria bacilliformis is a periodontal pathogen exacerbating periodontitis by inducing nitric oxide production
Source: Front Immunol. 2026 Jan 5;16:1735500. doi: 10.3389/fimmu.2025.1735500 (PMC12812723; doi:10.3389/fimmu.2025.1735500)
Supplement: Supplementary file 1 [file DataSheet1.docx]

***Neisseria bacilliformis* is a periodontal pathogen exacerbating periodontitis by inducing nitric oxide production**

**Running title:** *Neisseria bacilliformis* as a periodontal pathogen

Bo-Min Kim^1^, Yeonjin Lim^1^, Somin Park^1^, Jintaek Im^1^, Cheol-Heui Yun^2^, Kee-Yeon Kum^3^, Ok-Jin Park^1,*^, Seung Hyun Han^1,*^


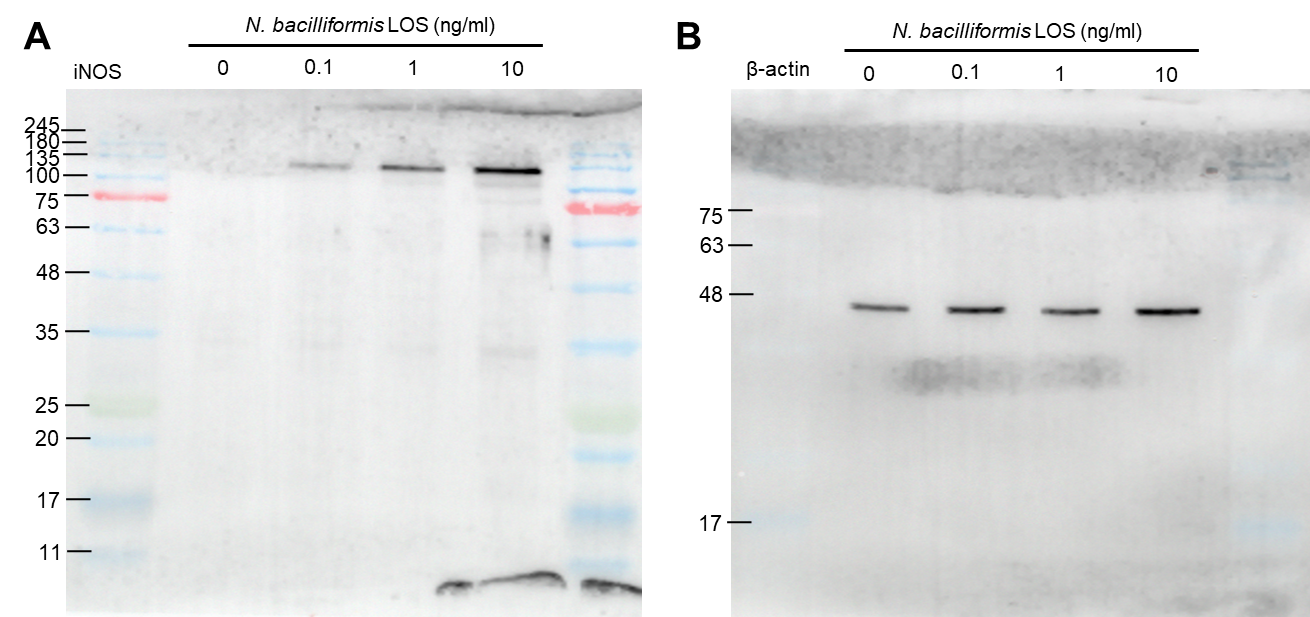


Supplementary Figure 1. Full-length Western blots with molecular weight markers showing iNOS expression induced by *N. bacilliformis* LOS in macrophages. RAW 264.7 cells (3 × 10⁵ cells/ml) were stimulated with Nb.LOS at 0.1, 1, or 10 ng/ml, or with Ec.LPS at 10 ng/ml for 24 h. Protein expression levels of iNOS and β-actin were assessed by Western blot analysis.


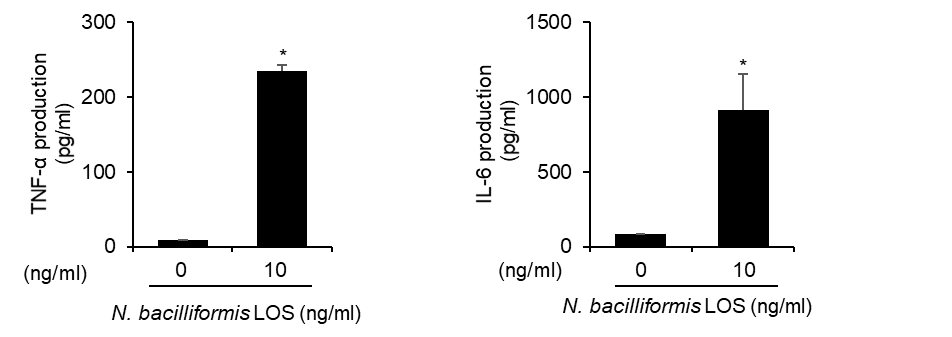


Supplementary Figure 2. *N. bacilliformis* LOS induced secretion of TNF-α and IL-6 in macrophages. RAW264.7 cells were seeded into 96-well plates at 200 μl of 3 × 10⁵ cells/ml per well and stimulated with the indicated stimuli for 24 h. Levels of TNF-α and IL-6 were quantified by ELISA.
