# Supplementary material for: Neisseria bacilliformis is a periodontal pathogen exacerbating periodontitis by inducing nitric oxide production
Source: Front Immunol. 2026 Jan 5;16:1735500. doi: 10.3389/fimmu.2025.1735500 (PMC12812723; doi:10.3389/fimmu.2025.1735500)
Supplement: Supplementary file 2 [file Table1.docx]

***Neisseria bacilliformis* is a periodontal pathogen exacerbating periodontitis by inducing nitric oxide production**

**Running title:** *Neisseria bacilliformis* as a periodontal pathogen

Bo-Min Kim^1^, Yeonjin Lim^1^, Somin Park^1^, Jintaek Im^1^, Cheol-Heui Yun^2^, Kee-Yeon Kum^3^, Ok-Jin Park^1,*^, Seung Hyun Han^1,*^

**Supplementary Table 1. Representative Ct values and ΔΔCt calculations for RT-qPCR analysis**

|  |  | *N. bacilliformis* LOS (ng/ml) | | |  |
| --- | --- | --- | --- | --- | --- |
|  | Non-treated | 0.1 | 1 | 10 | 10 ng/ml  *E. coli* LPS |
| iNOS (Ct) | 33.19768906 | 26.66792679 | 25.65485001 | 25.49855614 | 25.41975212 |
|  | 34.25143814 | 26.90921783 | 25.69792175 | 25.53739929 | 25.68305016 |
|  | 29.34218407 | 26.77734184 | 25.79582405 | 25.40642738 | 25.88167953 |
| GAPDH (Ct) | 18.12303925 | 18.25249481 | 18.19007111 | 18.31917191 | 17.6942997 |
|  | 17.94939995 | 17.92553711 | 18.01346397 | 18.22899437 | 17.67648125 |
|  | 17.48870277 | 17.97694206 | 18.02284241 | 18.23885536 | 17.58192444 |
| mean GAPDH | 17.854 | 18.052 | 18.075 | 18.262 | 17.651 |
| iNOS (ΔCt) | 15.34397507 | 8.61626879 | 7.57939084 | 7.23621559 | 7.76885033 |
|  | 16.39772415 | 8.85755984 | 7.62246259 | 7.27505875 | 8.03214836 |
|  | 11.48847008 | 8.72568385 | 7.72036489 | 7.14408684 | 8.23077774 |
| mean  iNOS (ΔCt) | 14.41005643 | 8.73317083 | 7.64073944 | 7.21845373 | 8.01059214 |
| iNOS (ΔΔCt) | 0.93391864 | -5.79378764 | -6.83066559 | -7.17384084 | -6.64120611 |
|  | 1.98766772 | -5.55249659 | -6.78759384 | -7.13499769 | -6.37790807 |
|  | -2.92158635 | -5.68437258 | -6.68969154 | -7.26596959 | -6.17927869 |
| 2^^-ΔΔCt^ | 0.52343466 | 55.47583754 | 113.82436403 | 144.39138334 | 99.81647877 |
|  | 0.25214618 | 46.93188794 | 110.47635698 | 140.55565470 | 83.16520051 |
|  | 7.57678786 | 51.42409464 | 103.22807207 | 153.91282002 | 72.46832740 |
| mean  2^^-ΔΔCt^ | 2.8 | 51.3 | 109.2 | 146.3 | 85.2 |
| STDEV | 4.1527855 | 4.273866636 | 5.416459307 | 6.877310958 | 13.781688 |
| T-test | 1 | 0.000147059 | 1.11886E-05 | 6.50356E-06 | 0.000581707 |
